# Arrhythmic Mitral Valve Prolapse Syndrome and Ventricular Arrhythmias: A Comprehensive Review and the Role of Catheter Ablation

**DOI:** 10.3390/jcdd11070218

**Published:** 2024-07-10

**Authors:** Ehsan Mahmoodi, Haris M. Haqqani

**Affiliations:** 1 Department of Cardiology, The Prince Charles Hospital, Brisbane, QLD 4032, Australia; ehsan.mahmoodi@health.qld.gov.au; 2 Faculty of Medicine, University of Queensland, Brisbane, QLD 4072, Australia

**Keywords:** ventricular arrhythmias, mitral valve prolapse, sudden cardiac death, catheter ablation

## Abstract

Mitral valve prolapse (MVP) affects 2–3% of the general population, and despite its benign prognosis overall, it is associated with sudden death in a small subset of patients. The term “arrhythmic MVP syndrome” (AMVPS) refers to the presence of frequent or complex ventricular arrhythmias, commonly reported in female patients with a stereotypical phenotype including bileaflet myxomatous disease, ECG repolarisation abnormalities in inferior leads, mitral annular disjunction, and significant fibrosis in the inferolateral LV and papillary muscles. Modern imaging technologies have led to the identification of new risk factors that have been implemented in recent risk stratification guidelines; however, screening for patients with MVP who are at risk of sudden cardiac death (SCD) remains challenging. In addition, there is a limited amount of data on the outcomes of different treatment approaches in AMVP and no specific indication for targeted or disease-modifying therapies within current guidelines. Potential arrhythmic substrates in patients with AMVP syndrome have been the subject of interest in previous studies, with areas consisting of fibrosis at the papillary muscle level and the Purkinje system. Premature ventricular contractions (PVCs) originating from these areas have been shown to play an important role as triggers for ventricular fibrillation and SCD in patients with AMVP. Catheter ablation has emerged as a potential treatment modality in patients with MVP and ventricular arrhythmias (VAs), targeting arrhythmic substrates and triggering PVC foci. The aim of this review is to explore the role of catheter ablation in treating patients with AMVP.

## 1. Introduction

Mitral valve prolapse (MVP) was first described by Criley et al. [1] as a type of mitral valve dysfunction leading to a late systolic murmur with a click, and it is characterised by the systolic superior displacement of one or both mitral leaflets at least two millimetres above the mitral annular plane [2]. It affects about 2–3% of the general population with an overall benign prognosis in the absence of hemodynamically significant mitral regurgitation [3]. The outcome of MVP is most often determined by the presence and severity of mitral regurgitation (MR), and early surgical intervention is the recommended approach in MVP with severe MR, having higher long-term survival compared with medical treatment [4].

However, in a subset of patients, MVP presents with ventricular arrhythmias and sudden cardiac death (SCD) even without hemodynamically significant MR, the so-called arrhythmic MVP (AMVP) syndrome [5]. Additionally, in those AMVPS patients who do have significant MR requiring intervention, SCD is well described even following successful surgical valve repair [6]. Estimating the precise incidence of SCD in MVP patients is challenging because of the lack of large cohorts and low overall event rate, but the prevalence of MVP in a series of autopsies of young adults with sudden arrhythmic death has been reported to be between 4% to 7% [7,8].

Sriram et al. [7] investigated the prevalence of MVP and its characteristics in 24 survivors of idiopathic out-of-hospital cardiac arrest. The authors characterised the malignant MVP phenotype by young women with bileaflet MVP, inferior biphasic or inverted T waves, and frequent premature ventricular contractions with the configuration of outflow tract alternating with papillary muscle or fascicular origin [7]. These clinical features were also demonstrated in the comprehensive study by Basso et al. [5], which assessed a consecutive series of patients presenting with complex ventricular arrhythmias or sudden cardiac death.

Since the delineation of the arrhythmic MVP phenotype, there has been an ongoing debate on its diagnosis and clinical implications, and despite the utilisation of multimodality approaches to identify MVP subsets at high arrhythmic risk [9], its natural history remains undetermined. In addition, there is a limited amount data on the long-term outcomes of various therapeutic approaches in patients with arrhythmic MVP without significant MR.

## 2. Arrhythmic Mitral Valve Prolapse Definition

Arrhythmic mitral valve prolapse (AMVP) syndrome is defined by the presence of MVP (with or without mitral annular disjunction), combined with frequent and/or complex VAs in the absence of any other well-defined arrhythmic substrate such as ischaemia, ventricular scar secondary to another defined aetiology, or channelopathy [9].

## 3. Arrhythmogenic Substrate in Arrhythmic Mitral Valve Prolapse

It is well known that severe MR in patients with MVP is an important predisposing factor for SCD and is associated with a high risk of ventricular arrhythmias [10]. The proarrhythmic mechanisms in MVP in the absence of mitral regurgitation are complex and the subject of active investigation [10,11].

The substrate underlying VAs in AMVP likely consists of an interplay between continuous mechanical papillary muscle stretch with chordal traction and friction at the inferobasal left ventricular (LV) wall, eventually leading to myocardial fibrosis [5]. In patients with primary MR, LV fibrosis is shown to be more prevalent in MVP than non-MVP cases, which may represent a unique pathophysiology beyond isolated volume overload in patients with MVP and a risk marker of arrhythmic events [12].

Nevertheless, a significant proportion of patients with MVP do not have clinical evidence of myocardial fibrosis, and SCD can occur earlier in the course of the disease. These patients may have more of a trigger-based arrhythmogenic mechanism such as traction of the papillary muscle unit and basal mid-myocardium activating cellular stretch receptors and causing complex VA [13].

In a prospective observational study by Miller et al. [14], patients with MVP and complex premature ventricular ectopy, and only mild to moderate MR, underwent VA characterisation and hybrid positron emission tomography (PET)/magnetic resonance imaging (MRI). The authors detected FDG uptake (PET) in the majority of patients, which was concordant with areas of late gadolinium enhancement (LGE, MRI). They suggested that chronic myocardial inflammation potentially contributes to the pathogenesis of myocardial injury in patients with MVP, even in the absence of significant MR. The utilisation of PET scan and its potential role in detecting myocardial inflammation in asymptomatic patients with MVP has been shown in other small studies, but its place in clinical MVP work-up and risk stratification remains to be defined [15].

### 3.1. Role of Papillary Muscles in Arrhythmogenesis

**Figure 1:**
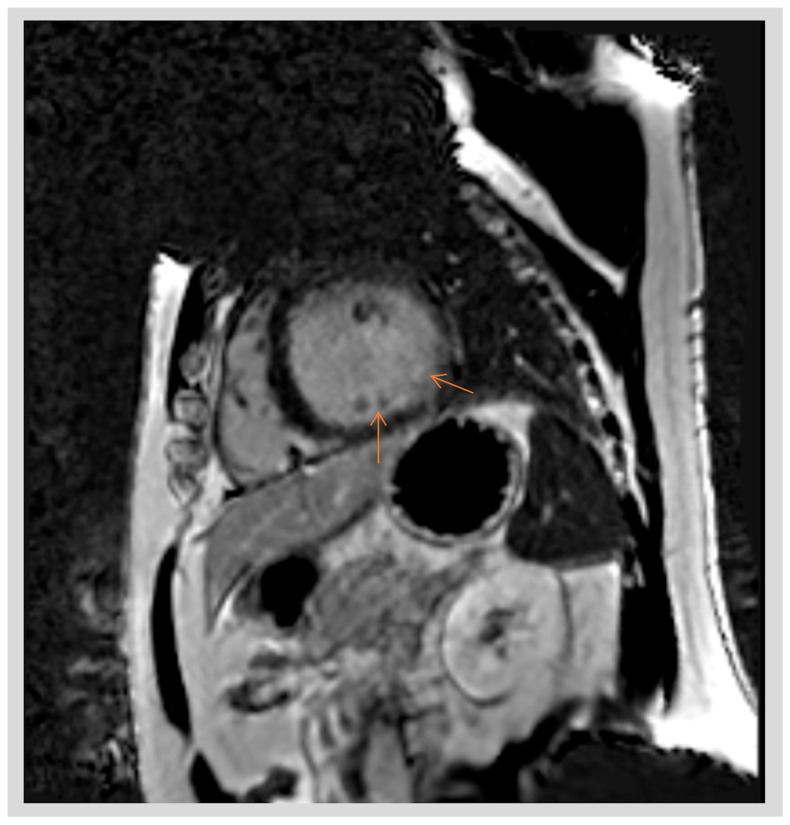
Cardiac MRI short-axis view of the left ventricle in a patient with AMVPS demonstrating areas of late gadolinium enhancement (LGE) affecting the posterolateral left ventricle and papillary muscle (arrows).

**Figure 2:**
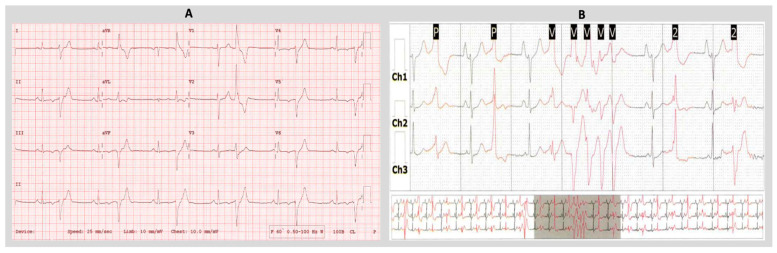
(**A**) Twelve-lead ECG in a patient with AMVPS, demonstrating sinus rhythm with ventricular bigeminy, PVCs with right bundle branch block (RBBB) morphology and superior axis, characteristic of posteromedial papillary muscle (PM) origin. (**B**) Three-channel Holter monitoring in a patient with AMVPS demonstrating frequent multifocal ventricular ectopies consisting of non-sustained runs and sustained ventricular bigeminy.

It has been shown in several studies that papillary muscle fibrosis may provide a substrate for VAs in patients with MVP [5,16]. Basso et al. [5] reported 650 autopsy cases of young adults with SCD, in which the authors assessed the MVP-associated arrhythmic risk based on the presence of LGE on cardiac MRI (Figure 1). In 43 patients with MVP, LV fibrosis at the PM level was detected at histology, and in 88% of patients, in the LV inferobasal wall. They also examined 44 living patients with MVP, both with (30) and without (14) complex VAs, using contrast-enhanced cardiac MRI. They reported a 93% rate of left ventricular LGE in MVP patients with complex ventricular arrhythmias (vs. 14% in the control group, *p* < 0.001), with an overlapping regional distribution with the histopathology findings in patients with MVP and SCD. Their study, for the first time, provided evidence of myocardial scarring targeting PMs and the adjacent inferobasal LV wall as the potential substrate for VAs in patients with MVP in the absence of significant MR [5]. This finding was consistent with the RBBB morphology of VAs arising from these areas (Figure 2) [17].

The presence of replacement fibrosis in the PM area associated with complex PVCs with PM origin in patients with MVP has been demonstrated in multiple previous observational studies [12,18,19]. Papillary muscle PVCs have been shown to play a role as triggers for ventricular fibrillation (VF) in patients with MVP [20].

### 3.2. Role of Purkinje Tissue in Arrhythmogenesis

The distal arborisations of the Purkinje system (PS), intimately associated with the papillary muscle, may represent a trigger for malignant VAs. It is likely that mechanical tension results in proarrhythmic changes in the papillary muscle electrophysiologic properties and that Purkinje fibres act as a trigger for VAs in areas with myocardial fibrosis. In a study by Syed et al. [21], it was shown that all patients with previous cardiac arrest had a Purkinje-origin PVC as the ventricular fibrillation trigger. The authors also demonstrated that Purkinje potentials at the site of ablation were associated with successful ablation. The authors concluded the Purkinje system plays a central role in triggering ventricular fibrillation in patients with arrhythmic MVP.

### 3.3. Role of Mitral Annular Disjunction in Arrhythmogenesis

Mitral annular disjunction (MAD) was first defined by Hutchins et al. [22] as a separation between the left atrial (LA) wall at the level of the MV junction and the LV free wall. They hypothesised that MAD could trigger mechanical stress on the leaflets leading to myxomatous degeneration. In a study by Perazzolo Marra et al. [23], the authors found that severe curling, which is defined as unusual systolic motion of the posterior mitral ring on the adjacent myocardium, results in mechanical stretch of the inferobasal left ventricular wall and papillary muscles, and subsequent myocardial hypertrophy and fibrosis. It was proposed that MAD plays a critical role in the cascade of morphofunctional abnormalities in MVP; hence, patients with MVP and annular abnormalities such as mitral annular disjunction and curling, require further arrhythmic risk stratification with contrast-enhanced cardiac MRI [23]. MAD is considered a potentially noteworthy finding in patients with arrhythmic MVP syndrome, and the presence of MAD in patients with AMVP was associated with more severe ventricular arrhythmias [24,25]. Moreover, arrhythmogenesis in MAD was reported irrespective of the presence of MVP [26]. However, population-level studies of MAD cast doubt on its implications [27].

## 4. Risk Stratification

There is a large population of asymptomatic patients with echocardiographically detectable MVP, and identifying the small subset of these patients who may be at high risk for developing severe VAs or SCD remains challenging. The recent Expert Consensus Statement of the EHRA [9] provided suggestions for arrhythmic risk stratification in patients with MVP. It is recommended that all patients with MVP should undergo SCD risk assessment utilising a focused clinical history, 12-lead ECG, extended ECG monitoring, and detailed echocardiography. T wave inversion (TWI) in the inferior and lateral leads have been independently associated with VAs in MVP patients [28]. The number of ECG leads with TWI has also been shown to correlate with higher arrhythmic risk and a higher degree of lateral diffuse fibrosis detected on cardiac MRI [29].

Mechanical traction of the posteromedial papillary muscle by myxomatous prolapsing leaflets in mid-systole results in sharp pulling of the adjacent posterobasal left ventricular wall, which is demonstrated as a spiked configuration of the lateral wall velocities (Pickelhaube sign) [30]. This echocardiographic finding serves as an early marker of mechanical stretch, even in the absence of LGE on cardiac MRI, and it is associated with an arrhythmic risk [30]. Other echocardiographic findings associated with an increased risk of developing VAs in patients with MVP include severe MR, bileaflet prolapse, a higher degree of prolapse, thickened mitral leaflets, and MAD [5,9,28,31,32,33,34].

The recent Expert Consensus Statement of the EHRA [9] recommended the use of implantable loop recorders (ILRs) in selected patients with high-risk features such as unexplained syncope with inconclusive Holter monitoring.

## 5. Treatment of Arrhythmic Mitral Valve Prolapse

### 5.1. Medical Therapy

There is a limited amount of data on the medical management of patients with arrhythmic MVP. Standard anti-arrhythmic drugs such as flecainide [35] appear to have a degree of efficacy in the suppression of ectopy in some patients, but randomised trials are lacking.

### 5.2. Implantable Cardioverter Defibrillator (ICD)

The guideline-directed indications for ICD also apply to patients with MVP and associated cardiomyopathy [36,37]. However, the primary prevention implantation of ICD in patients with arrhythmic MVP is a complex decision with no randomised trials to guide decision-making [9].

### 5.3. Catheter Ablation

**Figure 3:**
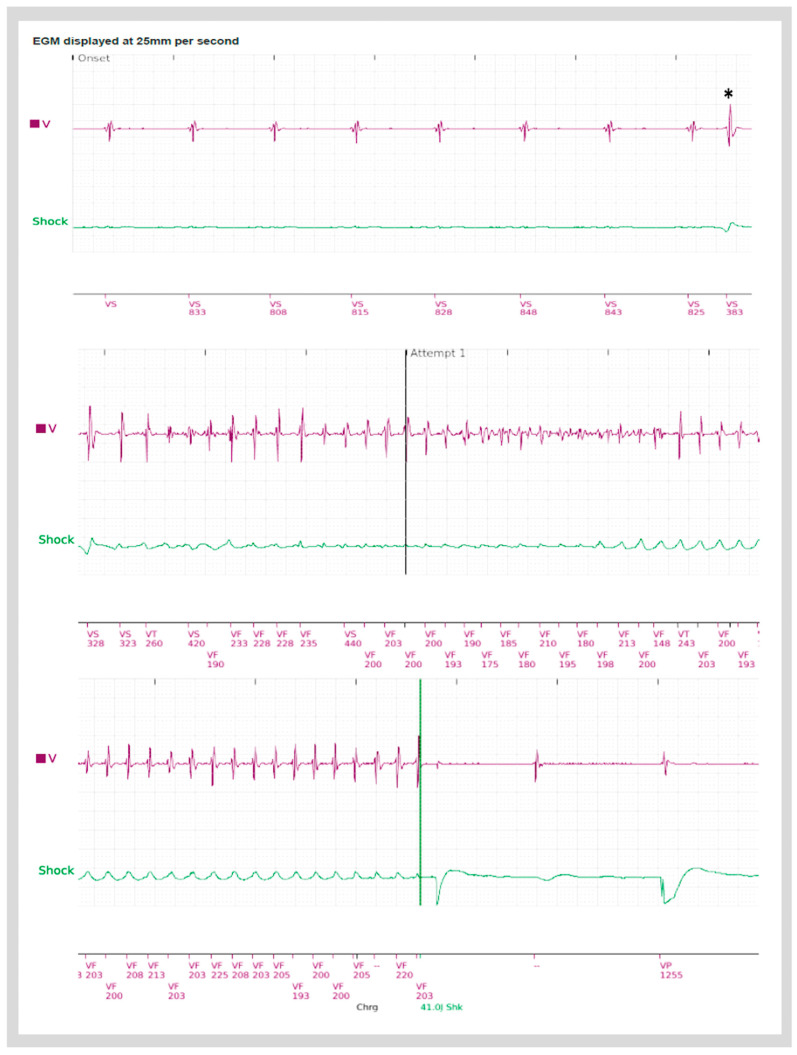
Electrograms from an implantable cardioverter defibrillator (ICD) in a patient with AMVPS demonstrating an episode of ventricular fibrillation, preceded by a premature ventricular complex (*), which was successfully terminated by one ICD shock.

There is a limited amount of data examining the role of catheter ablation (CA) in the treatment of VAs in patients with isolated MVP. Sudden ventricular fibrillation triggered by complex ventricular ectopy is considered to be the predominant mechanism of SCD in patients with AMVP syndrome (Figure 3) [21]. Thus, identification and elimination of the triggering PVC focus is an important treatment strategy with a reported long-term success rate of 60 to 84% [38,39,40]. Catheter ablation is also used in patients with MVP and symptomatic VAs refractory to medical treatment, recurrent appropriate ICD therapies, and PVC-mediated cardiomyopathy [21,37,39,41].

### 5.4. Outcomes of Catheter Ablation

Syed et al. [21] demonstrated the feasibility of presumed triggering PVC ablation in 14 consecutive patients with bileaflet MVP. Six patients had previous cardiac arrest and ICD, and eight patients had complex PVCs without previous cardiac arrest. All the patients had at least one PM or fascicular PVC focus. Purkinje-origin PVC was the VF trigger for all the patients with previous cardiac arrest, and it was the dominant PVC origin in five of eight patients without previous cardiac arrest. Acute procedural success was seen in 12 of 14 patients. At the 478-day follow-up, two patients in the cardiac arrest group received appropriate ICD shocks and six patients required a redo procedure for recurrent symptomatic arrhythmias. The authors also reported procedural proarrhythmic potential in patients who developed VF during or immediately after the ablation of targets exhibiting Purkinje potentials. The transient increased risk of developing malignant VAs during and immediately after radiofrequency ablation of Purkinje tissue has previously been reported [42,43]. The authors concluded that catheter ablation can effectively reduce symptomatic PVC burden and appropriate ICD shocks.

In a study by Lee et al. [41], 152 consecutive patients underwent catheter ablation for focal VAs. They described the prevalence of MVP in LV papillary muscle VAs and examined the outcomes of CA in LV papillary muscle VAs versus other focal sites and LV papillary muscle VAs by the presence of MVP and cardiomyopathy. Twenty-three patients underwent papillary muscle VA ablation, nine of which (39%) had MVP compared with none of the one-hundred twenty-nine (0%) patients at other sites (*p* < 0.001). They showed comparable acute procedural success in 60% and 80% of those with and without MVP, respectively (*p* = 0.28). In the patients with cardiomyopathy, the median LV ejection fraction (LVEF) improved from 40% to 54% following catheter ablation (*p* = 0.007). The authors concluded that despite a strong association between LV papillary muscle VAs and MVP, the presence of MVP did not adversely impact acute or medium-term outcomes of ablation, but they acknowledged the study was likely underpowered to exclude a difference in outcome with MVP. They also demonstrated the efficacy of catheter ablation in improving LV systolic function in papillary muscle ectopy-mediated cardiomyopathy.

**Figure 4:**
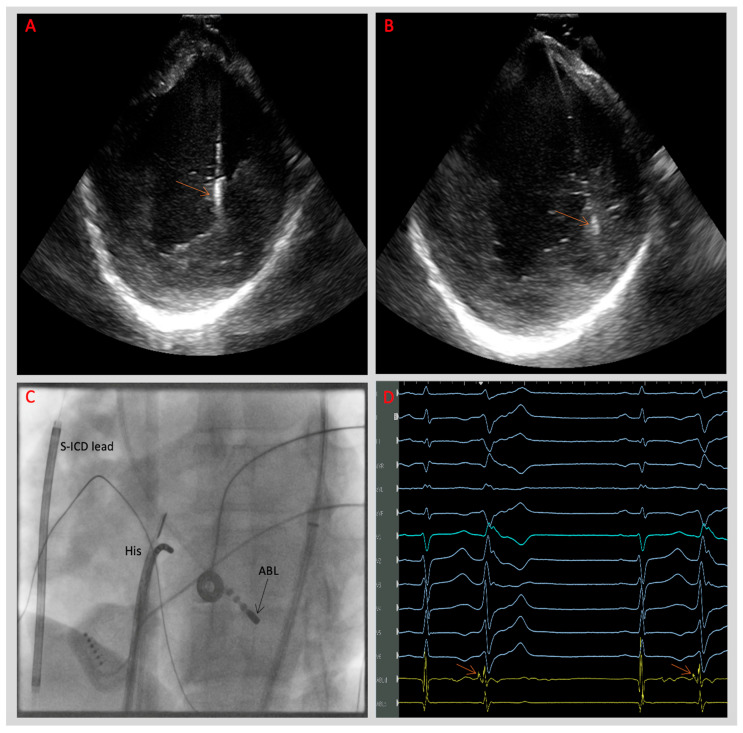
(**A**,**B**) Intracardiac echocardiography (ICE) views of the left ventricle demonstrating ablation catheter contact (arrows) with posteromedial papillary muscle (PM) during radiofrequency (RF) energy delivery in a patient with AMVPS. (**C**) Fluoroscopy in the same patient demonstrating an ablation catheter tip (black arrow, ABL) at the PM level. (**D**) Electrogram demonstrating the site of successful ablation in a patient with AMVPS with frequent PVCs originating from posteromedial papillary muscle (RBBB morphology with superior axis) preceded by Purkinje potentials (arrows on the ablation catheter [ABL] channel). (His: Hexapolar catheter at the His bundle level, S-ICD: subcutaneous implantable cardioverter defibrillator).

Papillary muscles are mobile endocavitary structures that can limit catheter stability and catheter tissue contact force, making papillary muscle VA ablation challenging [44]. In patients with MVP, chronic mechanical stretch can result in papillary muscle hypertrophy, which can also lead to a technically difficult ablation by protecting the deep arrhythmogenic foci from endocardial ablation [20]. In addition, the papillary muscle structure may allow for a single arrhythmic focus with multiple exit points, producing different QRS morphologies. And of course, scarred papillary muscles may be able to support multiple potential re-entrant VAs. Hence, even with detailed activation data and an excellent pacemap, papillary muscle PVC ablation usually requires extensive ablation along the papillary muscle [42,45]. Lee et al. [41] used a phased array intracardiac echocardiography (ICE) probe for visualisation of the LV papillary muscles (Figure 4, Supplementary Materials). Despite the challenging nature of papillary muscle VA ablation, their medium outcome was comparable to the outcomes of patients who underwent ablation of VAs from other sites. Moreover, extensive ablation in this area did not result in acute or delayed valvular dysfunction.

In another study by Enriquez et al. [39], the authors investigated the electrophysiological substrate and outcomes of catheter ablation in 25 patients with MVP and papillary muscle PVCs. Four patients presented with PVC-triggered VF. Nine patients had cardiac MRI, and LGE was demonstrated in four of them (three of whom had focal PM LGE). Eleven patients underwent detailed LV voltage mapping, three of whom demonstrated bipolar voltage abnormalities isolated to the inferior and inferolateral walls. The authors reported a successful ablation in the majority with complete PVC elimination in 19 (76%) patients and a significant reduction in PVC burden (from 20.4% ± 10.8% to 6.3% ± 9.5%, *p* = 0.001) in two (8%) patients. LVEF improved in five of six patients with impaired LV systolic function following successful ablation, suggesting the likely aetiology of PVC-mediated cardiomyopathy, which is in keeping with the study by Lee et al. [41] with subsequent normalisation of LV function in the majority of patients after successful papillary muscle foci ablation. Enriquez et al. [39] also emphasised the routine use of ICE and its role in overcoming the challenges associated with variable PM morphology and ensuring adequate contact with the ablation target. Cryoablation was not used in patients in their study, similar to the study by Lee et al. [41], where the majority of the patients had radiofrequency ablation.

Rivera et al. [46] compared the outcomes of catheter ablation with either radiofrequency (RF) or cryoenergy in 21 patients with drug-refractory LV papillary muscle VAs. It was demonstrated that using cryoablation was associated with better catheter stability and a higher success rate compared with radiofrequency ablation. They updated these findings in another study by utilising contact force sensing (CFS) catheters and advanced electroanatomical mapping (EAM) systems [47]. The outcomes of catheter ablation of PM VAs were compared using different energy sources including CFS RF, Cryoablation (CRYO), and non-CFS RF. It was shown that both the CRYO and CFS RF approaches were associated with high success rates, as opposed to a 4.6-fold increased risk of recurrence of clinical arrhythmia when using non-CFS RF. Intracardiac echo-facilitated 3D electroanatomic mapping was shown to be strongly associated with procedural success, and the routine use of this technique has been previously proposed to improve PM VA ablation outcomes [48].

Although Purkinje- and PM-origin PVCs are the predominant triggers for ventricular fibrillation in patients with MVP, late recurrence could be due to different arrhythmic foci, which necessitate long-term patient follow-up. Marano et al. [38] assessed the long-term outcome of catheter ablation in 15 patients with bileaflet MVP and complex ventricular ectopy. Over a median follow-up of nine years, 5 of 15 (33%) patients developed haemodynamically significant VT/VF requiring ICD implantation. All five patients who developed VT/VF had complex PVCs and inducible sustained VT prior to index ablation. Three of the five patients required redo ablations for a new dominant focus of ventricular arrhythmia, which likely represents the progressive nature of ventricular arrhythmias in patients with MVP. These outcomes suggest that inducible sustained VAs and complex multifocal ventricular ectopies may indicate a more diffuse disease process associated with a higher risk of VA recurrence [38].

Catheter ablation in patients with MVP and VAs has evolved over the years, and multiple factors have led to higher procedural success rates [49]. These include the utilisation of intracardiac echocardiography (ICE), advanced electroanatomic mapping systems, and mapping with contact force catheters. Additionally, the use of half-normal saline irrigation allows for potential increases in lesion depth after high-power RF current delivery [39,40,50]. Despite all these advances in ablation techniques, catheter ablation in patients with MVP and VAs remains challenging even in high-volume centres, and more studies are required in order to improve its long-term outcomes.

### 5.5. Effect of Mitral Valve Intervention in Arrhythmic Mitral Valve Prolapse

Mitral valve surgery may reduce the VA burden in arrhythmic MVP; however, the available data are limited to small case series and isolated case reports [51,52,53,54,55]. The efficacy of mitral valve surgery in reducing VA burden in MVP is presumably attributed to reduced mechanical stretch on the papillary muscles and altering the LV remodelling process [56].

Benito-González et al. [57] evaluated the VA burden before and after percutaneous mitral valve repair (PMVR) in 34 heart failure patients with ICD and functional MR. It was reported that successful mitral valve repair was associated with reduced arrhythmic burden and ICD therapies. In another study by Vohra et al. [58], the successful delivery of targeted papillary muscle cryoablation at the time of mitral valve repair was demonstrated. They assessed the outcome of direct visually guided surgical cryoablation of papillary muscles at the time of elective mitral valve surgery in three patients with MVP, moderate to severe MR, and recurrent ICD therapies for drug-refractory malignant VAs. During the follow-up period, there was no recurrence of VAs in any of the three patients, and cryoablation lesions did not result in valvular malfunction. Further studies on larger cohorts of patients are clearly required to define the efficacy, safety, and long-term outcomes of this combined procedure.

Although surgical intervention in patients with MVP and severe MR reduces the VA burden, the role of surgical intervention in patients with arrhythmic MVP and moderate MR remains controversial because of inconsistent results in the available studies [28,59,60]. Defining clear thresholds for surgical intervention in patients with AMVP syndrome is an important goal the field faces and will necessitate large prospective controlled studies.

## 6. Conclusions

Arrhythmic MVP syndrome likely represents a progressive disorder with a variety of arrhythmic manifestations. These include focal isolated PVC, sometimes with associated cardiomyopathy, sustained VT, and ectopy triggering VT/VF. The papillary muscles and adjacent Purkinje foci represent the commonest arrhythmogenic sites and the origin of these VAs. Catheter ablation can be effective in treating symptomatic PVCs, PVC-mediated cardiomyopathy, and PVC-triggered VT/VF. Although utilising ICE, cryoablation, and contact force sensing can improve the success rate of catheter ablation of the papillary muscle in patients with MVP, it can be challenging because of a range of anatomic and technical factors. Additional well-designed prospective trials are required to further define the efficacy and long-term outcomes of catheter ablation in AMVP patients with VAs.

## Data Availability

Not applicable.

## Supplementary Materials

The following supporting information can be downloaded at: https://www.mdpi.com/article/10.3390/jcdd11070218/s1, Videos S1 and S2: Intracardiac echocardiography (ICE) views of the left ventricle demonstrating ablation catheter contact with posteromedial papillary muscle (PM) during radiofrequency (RF) energy delivery in a patient with AMVPS.
